# Untreated, Impacted Canine Leading to Root Resorption of the Central Incisor: A Case Report of Orthodontic Treatment With Canine Substitution

**DOI:** 10.7759/cureus.106124

**Published:** 2026-03-30

**Authors:** Shuhao Xu, Bo Zheng, Xiaolong Li, Wei Li, Xi Zhao

**Affiliations:** 1 Department of Stomatology, Deyang People's Hospital, Deyang, CHN; 2 Department of Stomatology, Deyang People’s Hospital, Deyang, CHN; 3 Pulmonary and Critical Care Medicine, Deyang People's Hospital, Deyang, CHN

**Keywords:** canine substitution, ectopic canines, impacted canines, orthodontic traction, root resorption, surgical exposure

## Abstract

Among impacted teeth, maxillary canines are the most frequently impacted after third molars. Untreated impacted canines may lead to complications such as root resorption of adjacent teeth. Therefore, early diagnosis and treatment of impacted canines are very important. The treatment methods, such as creating space for eruption, extraction of the deciduous canine, and surgical exposure with orthodontic traction, should be promptly employed to guide the canine to erupt into its normal position. We reported a case of an 11-year-old girl with maxillary canine impaction leading to root resorption of the central incisor. Unfortunately, a tendency toward canine impaction had been detected when she was six years old, but timely intervention was not provided, ultimately resulting in root resorption and extraction of the central incisor. Through orthodontic extraction camouflage treatment, the lateral incisor ultimately substituted for the central incisor, achieving a relatively satisfactory treatment outcome. This case once again underscores the importance of early treatment for impacted canines. But when the incisor root resorption is too severe to retain the tooth, orthodontic treatment with canine substitution is also an acceptable, compromised treatment option.

## Introduction

A canine that deviates from its normal eruption path can be referred to as an ectopic canine [1]. Ectopic canines may present as labial, palatal, and/or mesiodistal versions. Ectopic canines can potentially lead to impaction. Among impacted teeth, maxillary canines are the most frequently impacted after third molars, and the prevalence of maxillary canine impaction has been reported to be 0.2%-2.8% in the general population [2]. Statistical results from Asia indicate that labial impactions are more common than palatal impactions [3]. Conversely, among Caucasian populations, palatal impaction is more common than buccal impaction [4]. Mandibular impacted canines are relatively rare, with an incidence rate 2 to 20 times lower than that of maxillary canines [5]. There is a significant gender difference in canine impaction, with a higher incidence in females [6].

Canines play a significant role in both the function and aesthetics of the oral and maxillofacial region [7]. Located at the corners of the dental arch, canines are essential for bearing occlusal forces, supporting the corners of the mouth, and contributing to facial aesthetics. In particular, maxillary canines are associated with the fullness of the midface. If a canine is missing or ectopically positioned on the palatal side, it may lead to the collapse of the dental arch, affecting the fullness of the middle third of the face and resulting in a sunken mouth and aged facial appearance. Furthermore, failure to timely treat impacted or ectopic canines may lead to complications such as root resorption of adjacent teeth, displacement of adjacent teeth, loss of arch length, malocclusion, and the formation of dentigerous cysts, causing permanent damage to the integrity of the dentition and oral function [8]. According to one previous study, 9.4% ectopic canines cause root resorption of adjacent incisors [9]. However, Asians are more prone to root resorption of adjacent teeth caused by impacted canines [10]. Therefore, early detection and timely treatment of impacted canines are of great significance in preventing these complications.

When an impacted canine has caused severe root resorption of an incisor, various factors such as age, chief complaint, and the severity of resorption need to be considered [11]. Although some reports have indicated that retaining an incisor with root resorption remains a feasible treatment option, orthodontic movement does not exacerbate mobility or discoloration of the adjacent tooth with severe root resorption caused by an impacted tooth, and splinting is also unnecessary after treatment; moreover, the incisor with root resorption can maintain pulp vitality even without root canal treatment [12,13]. Due to the poor long-term prognosis, as well as concerns regarding treatment time and cost, it is generally recommended to extract the incisor with severe resorption and proceed with treatment through canine substitution or implant restoration [14].

Early diagnosis and monitoring of maxillary canines are crucial for preventing their impaction. It is recommended to conduct a comprehensive clinical examination before the age of 9-10 years [15], including visual inspection and palpation of the buccal sulcus and palatal mucosa, to observe whether there is a lack of buccal bulge but a presence of palatal bulge. At this stage, the root development of the canine should have reached one-half to two-thirds of its full length. Regular check-ups are advised, with annual longitudinal panoramic radiographs for monitoring, to carefully observe the development of the dentition during the mixed dentition period and to intervene at the appropriate time.

For already impacted maxillary canines, treatment methods such as creating space for eruption, extraction of the deciduous canine, and surgical exposure with orthodontic traction should be promptly employed to guide the canine to erupt into its normal position, and autotransplantation is also a feasible treatment option [16]. Given the importance of maxillary canines, the extraction of an impacted canine should only be considered when the tooth germ is severely abnormal, inverted, severely rotated, or when the eruption path deviates so significantly from the normal range that the success rate of orthodontic traction is low [17].

This case report described an untreated impacted maxillary canine in an 11-year-old girl, which ultimately led to root resorption and extraction of the central incisor. Through orthodontic extraction treatment, combined with surgical exposure and orthodontic traction of the impacted canine, after a long treatment period of 36 months, the maxillary canine was successfully guided into the lateral incisor position, and the lateral incisor was reshaped to mimic the central incisor, achieving a relatively ideal treatment outcome. This study was performed in line with the principles of the Declaration of Helsinki. Due to the retrospective nature of this case report, formal ethical approval was not required by the Ethics Committee of Deyang People’s Hospital. However, written informed consent was obtained from the patient‘s parents for the publication of this case report and any related clinical data. All identifying information has been removed to ensure patient anonymity.

## Case presentation

**Figure 1:**
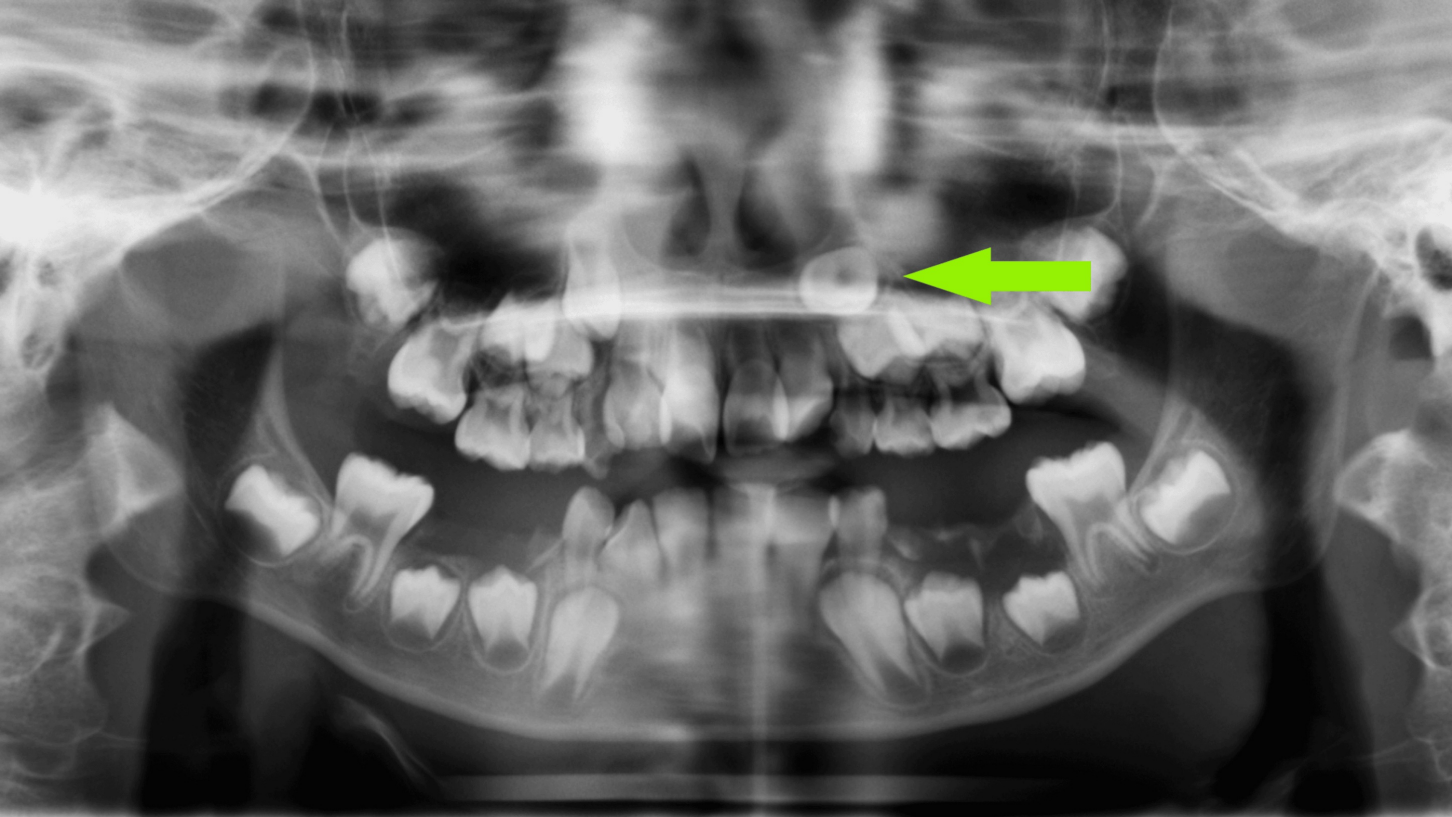
Panoramic radiograph taken five years before the start of treatment The tooth germ of tooth 23 was abnormally oriented and at risk of impaction (green arrow).

An 11-year-old girl was referred to our department due to root resorption of the maxillary central incisor caused by an impacted maxillary canine. Five years ago, the patient's parents brought her to the department of pediatric dentistry for the extraction of primary teeth that were not worth preserving. At that time, a panoramic radiograph (Figure 1) revealed that the tooth germ of tooth 23 was abnormally oriented and at risk of impaction. Further consultation at the orthodontic clinic after completion of caries treatment was recommended, but the parents did not follow up or seek treatment. The patient had no significant history of systemic disease and reported no drug allergy.

**Figure 2:**
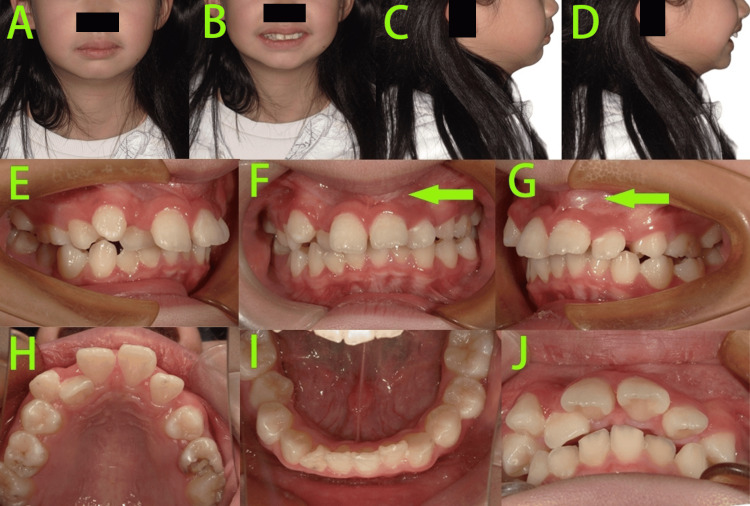
Pre-treatment photos A-D: Facial photos showed a convex profile, mandibular retrusion, normal mandibular angle, normal nasolabial angle, deep mentolabial sulcus, slight facial asymmetry, a short lower third of the face, and a generally normal lip-to-tooth relationship. E-J: Intraoral photos revealed that the patient was in the late mixed dentition stage, with retained primary teeth 55 and 65 showing evidence of caries, tooth 23 was not visible intraorally and lacked sufficient space for eruption, palpation of the labial apical region of tooth 21 revealed a bulge caused by the crown of tooth 23 (green arrow).

Facial examination (Figures 2A-2D) showed a convex profile, mandibular retrusion, normal mandibular angle, normal nasolabial angle, deep mentolabial sulcus, slight facial asymmetry, a short lower third of the face, and a generally normal lip-to-tooth relationship. Intraoral examination (Figures 2E-2J) revealed that the patient was in the late mixed dentition stage, with retained primary teeth 55 and 65 showing evidence of caries. Tooth 23 was not visible intraorally and lacked sufficient space for eruption. Palpation of the labial apical region of tooth 21 revealed a bulge caused by the crown of tooth 23. The bilateral molars exhibited a distal cusp-to-cusp relationship. Moderate crowding was observed in the maxillary arch, while the mandibular arch showed mild crowding. Additionally, a deep overbite and deep overjet were noted in the anterior teeth.

**Figure 3:**
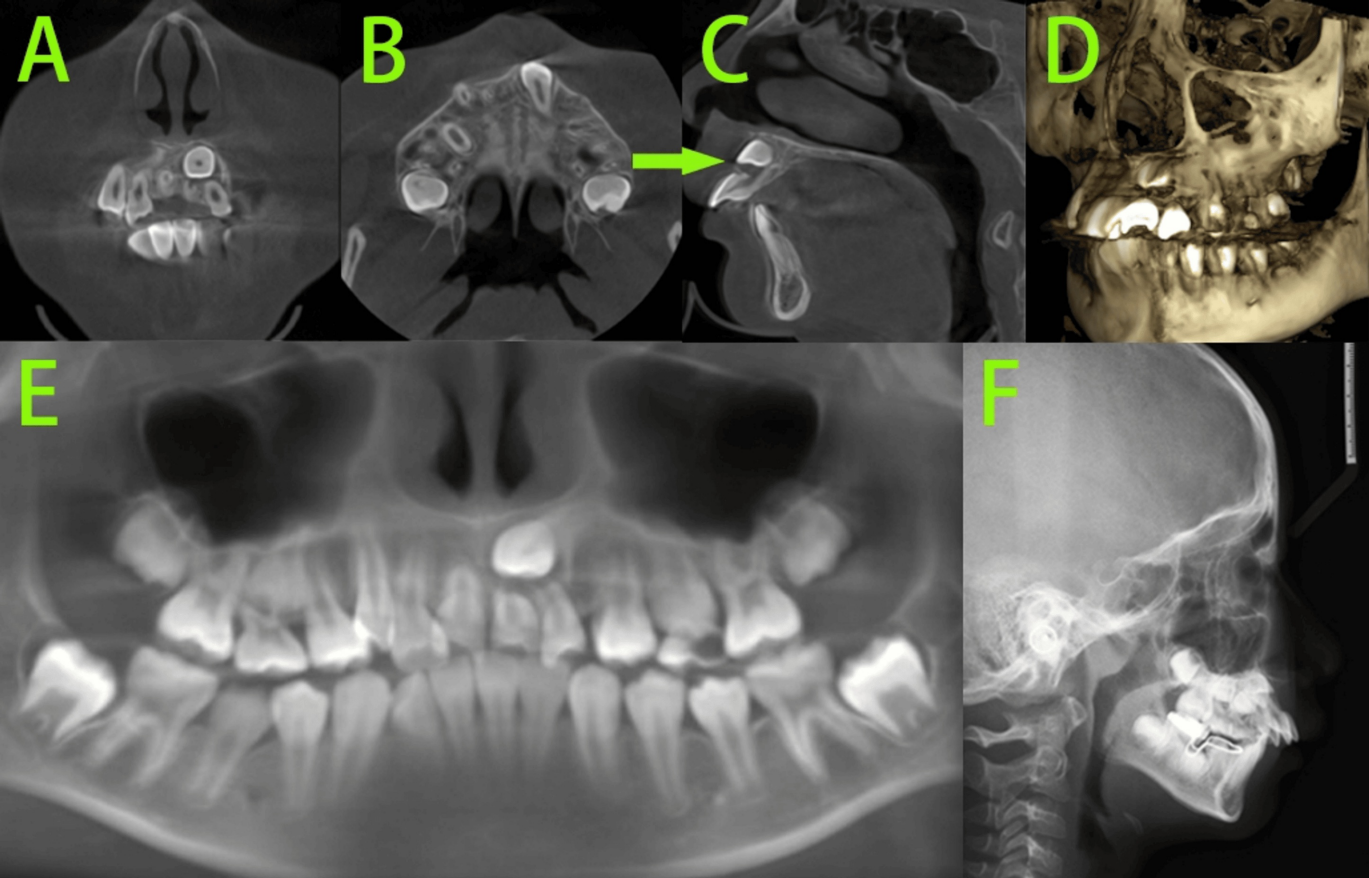
Pre-treatment radiographs A-C: CBCT revealed root resorption was observed in the retained primary teeth 55 and 65, tooth 23 was ectopic and impacted in the apical region of tooth 21, and significant root resorption of tooth 21 was already evident (green arrow). D: CBCT 3D reconstruction reveals that tooth 23 is impacted on the labial side of the root of tooth 21. E: The CBCT-synthesized panoramic image showed mesial inclination of the axis of tooth 23. F: lateral cephalometric radiograph taken at another hospital six months ago indicated that the patient was in the pre-peak growth period, a skeletal Class II pattern with mandibular retrusion, a normodivergent facial angle, an average growth pattern, proclination of the maxillary incisors, and upright mandibular incisors.

Pre-treatment cone-beam computed tomography (CBCT) (Figures 3A-3E) showed no abnormalities in the number of permanent tooth germs, and root resorption was observed in the retained primary teeth 55 and 65. Tooth 23 was ectopic and impacted in the apical region of tooth 21, with its axis exhibiting mesial inclination and labial tipping. Significant root resorption of tooth 21 was already evident. A lateral cephalometric radiograph taken at another hospital six months ago (Figure 3F) showed no significant narrowing of the upper airway, cervical vertebral maturation stage 2 (CVS 2), indicating that the patient was in the pre-peak growth period. Cephalometric analysis (Table 1) indicated a skeletal Class II pattern with mandibular retrusion, a normodivergent facial angle, an average growth pattern, proclination of the maxillary incisors, and upright mandibular incisors.

**Table 1:** Pre-treatment cephalometric measurements

Cephalometric parameters	Measured value	Reference value
SNA(°)	81.7	83.0±4.0
SNB(°)	73.7	80.0±4.0
ANB(°)	8.0	3.0±2.0
Wits(mm)	6.6	0.0±2.0
S-Go/N-Me(%)	62.1	64.0±2.0
FMA(°)	29.2	26.0±4.0
U1-SN(°)	118.9	106.0±6.0
IMPA(°)	91.9	97.0±6.0

Diagnosis: Caries of primary teeth 55 and 65; Skeletal Class II malocclusion with mandibular retrusion; Normodivergent facial angle, average growth pattern; Impaction of tooth 23, root resorption of tooth 21; Dental crowding, deep overbite, and increased overjet. Considering the significant root resorption of tooth 21, combined with the patient’s skeletal Class II malocclusion and dental crowding, orthodontic extraction camouflage treatment might be considered. The extraction pattern would involve removing tooth 21, along with the maxillary first premolar and mandibular second premolars in the other quadrants, to correct the distal molar relationship and improve anterior overjet and overbite. The advantage of this approach was that it avoided the need for prosthetic restoration after eventual loss of tooth 21; however, the disadvantage was a less satisfactory anterior aesthetic outcome. The alternative approach was to distally retract tooth 23, consult the endodontists to preserve tooth 21 if possible, and utilize maxillary expansion combined with functional appliance therapy to promote mandibular growth. The advantage of this approach was the potential for greater mandibular growth and improved anterior aesthetics, while the disadvantage was the poor long-term prognosis of tooth 21, which might ultimately require prosthetic restoration. After detailed communication with the patient's parents, they were willing to choose the camouflage treatment with extractions. Therefore, the final comprehensive orthodontic treatment plan for this patient was as follows: oral hygiene instruction; extraction of teeth 55, 65, 14, 21, 35, and 45; surgical exposure and orthodontic traction of tooth 23 to the distal, mesialization of tooth 22; leveling and alignment of the maxillary and mandibular arches; retraction of the maxillary anterior teeth; closure of extraction spaces; achieving a Class I molar relationship; establishing normal anterior overbite and overjet; creating mesiodistal space distal to tooth 22 for subsequent composite resin reshaping of tooth 22 to simulate a central incisor (to be restored with veneers in adulthood); final occlusal refinement; and retention.

**Figure 4:**
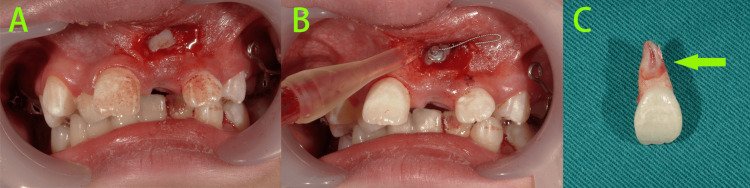
Intraoral photos at the start of treatment A-B: Teeth 55, 65, and 21 were extracted under local anesthesia, and surgical exposure of tooth 23 was performed to expose the crown and bond a traction device. C: The extracted tooth 21 showed significant resorption of the labial root (green arrow).

After thoroughly explaining the patient's condition and treatment plan to the parents and obtaining their informed consent, treatment was initiated. First, a transpalatal arch (TPA) with a soldered hook for traction was fabricated to reinforce anchorage. Simultaneously, teeth 55, 65, and 21 were extracted under local anesthesia, and surgical exposure of tooth 23 was performed to expose the crown and bond a traction device (Figures 4A, 4B). The extracted tooth 21 showed significant resorption of the labial root (Figure 4C).

**Figure 5:**
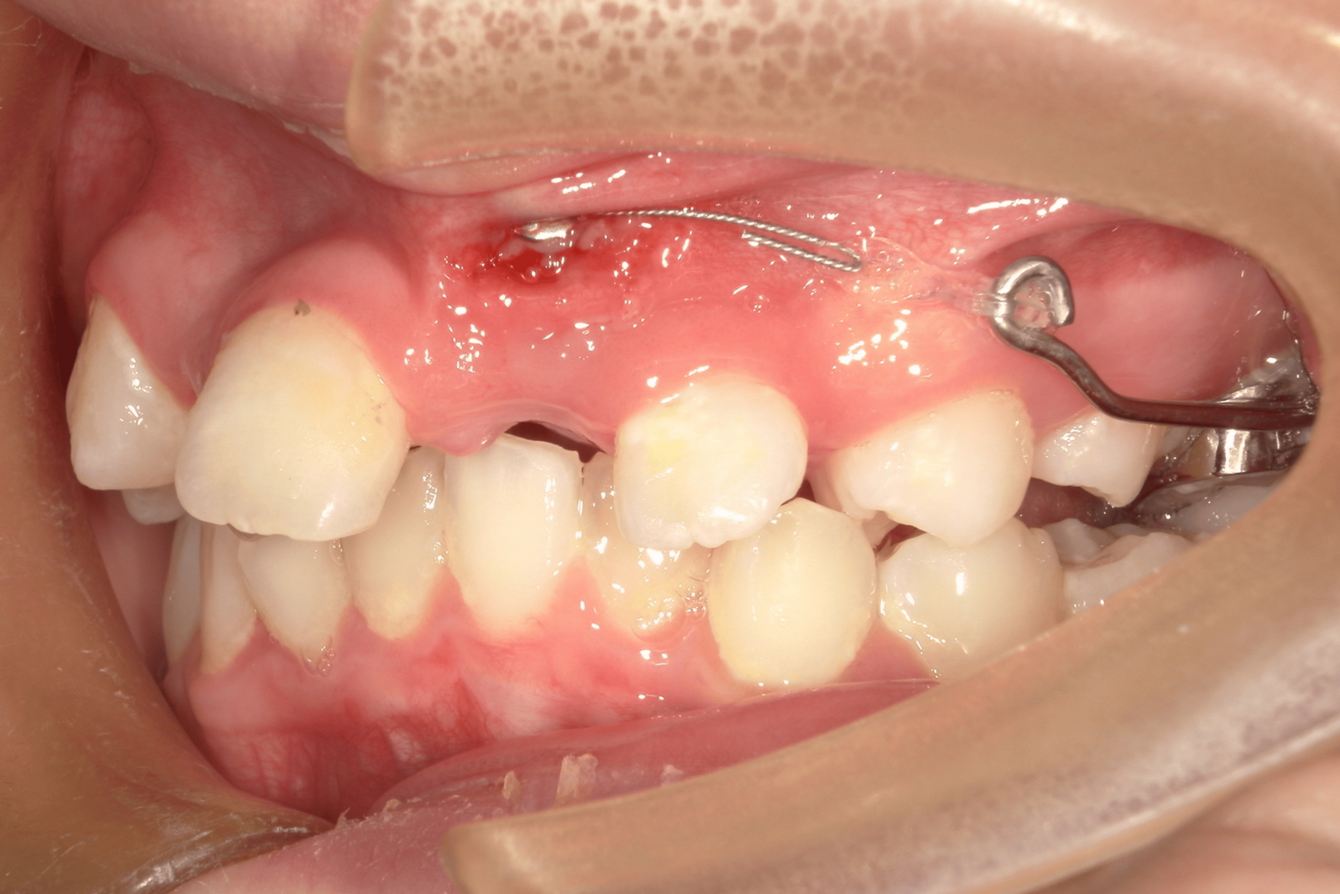
Intraoral photos one week after surgical exposure of tooth 23 Distal traction of tooth 23 was initiated.

One week after surgical exposure (Figure 5), distal traction of tooth 23 was initiated.

**Figure 6:**
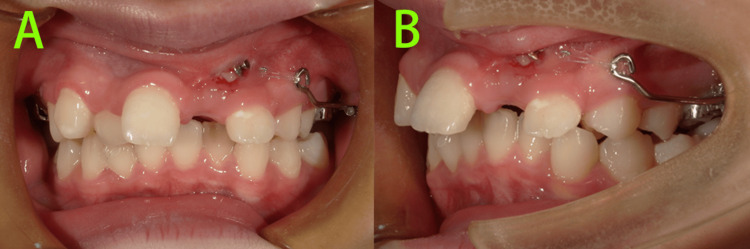
Intraoral photos at three months of treatment A-B: The crown of tooth 23 was partially exposed in the oral cavity, and distal traction of tooth 23 was continued.

Three months after treatment initiation (Figure 6), the crown of tooth 23 was partially exposed in the oral cavity, and distal traction of tooth 23 was continued.

**Figure 7:**
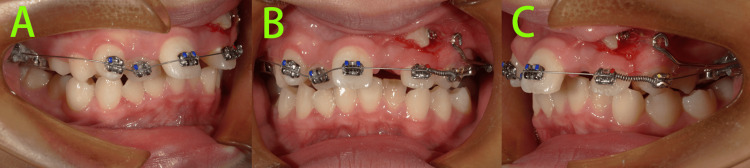
Intraoral photos at six months of treatment A-C: Distal traction of tooth 23 was continued, and a fixed sectional orthodontic appliance was bonded in the maxillary arch to align the anterior teeth and mesialize tooth 22.

At six months of treatment (Figure 7), the crown of tooth 23 was more exposed intraorally. Teeth 15 and 25 had fully erupted. Distal traction of tooth 23 was continued, and a fixed sectional orthodontic appliance (HX metal brackets, Shinye Biotech, China) was bonded in the maxillary arch to align the anterior teeth and mesialize tooth 22.

**Figure 8:**
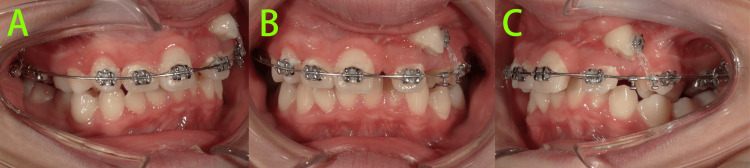
Intraoral photos at 10 months of treatment A-C: Mesialization of tooth 22 was continued, along with occlusal traction of tooth 23.

At 10 months of treatment (Figure 8), tooth 23 had been successfully moved distally into position, and the maxillary anterior teeth were aligned. The TPA was retained and the traction hook was ground off. Mesialization of tooth 22 was continued, along with occlusal traction of tooth 23.

**Figure 9:**
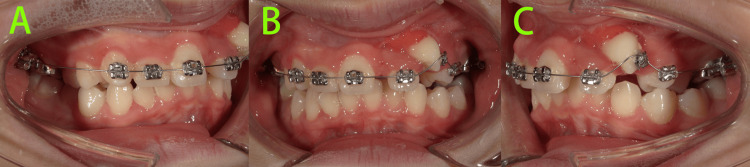
Intraoral photos at 12 months of treatment A-C: A bracket was bonded on tooth 23 and a 0.012-inch NiTi archwire was used to align tooth 23.

At 12 months of treatment (Figure 9), we bonded a bracket on tooth 23 and used a 0.012-inch NiTi archwire to align tooth 23.

**Figure 10:**
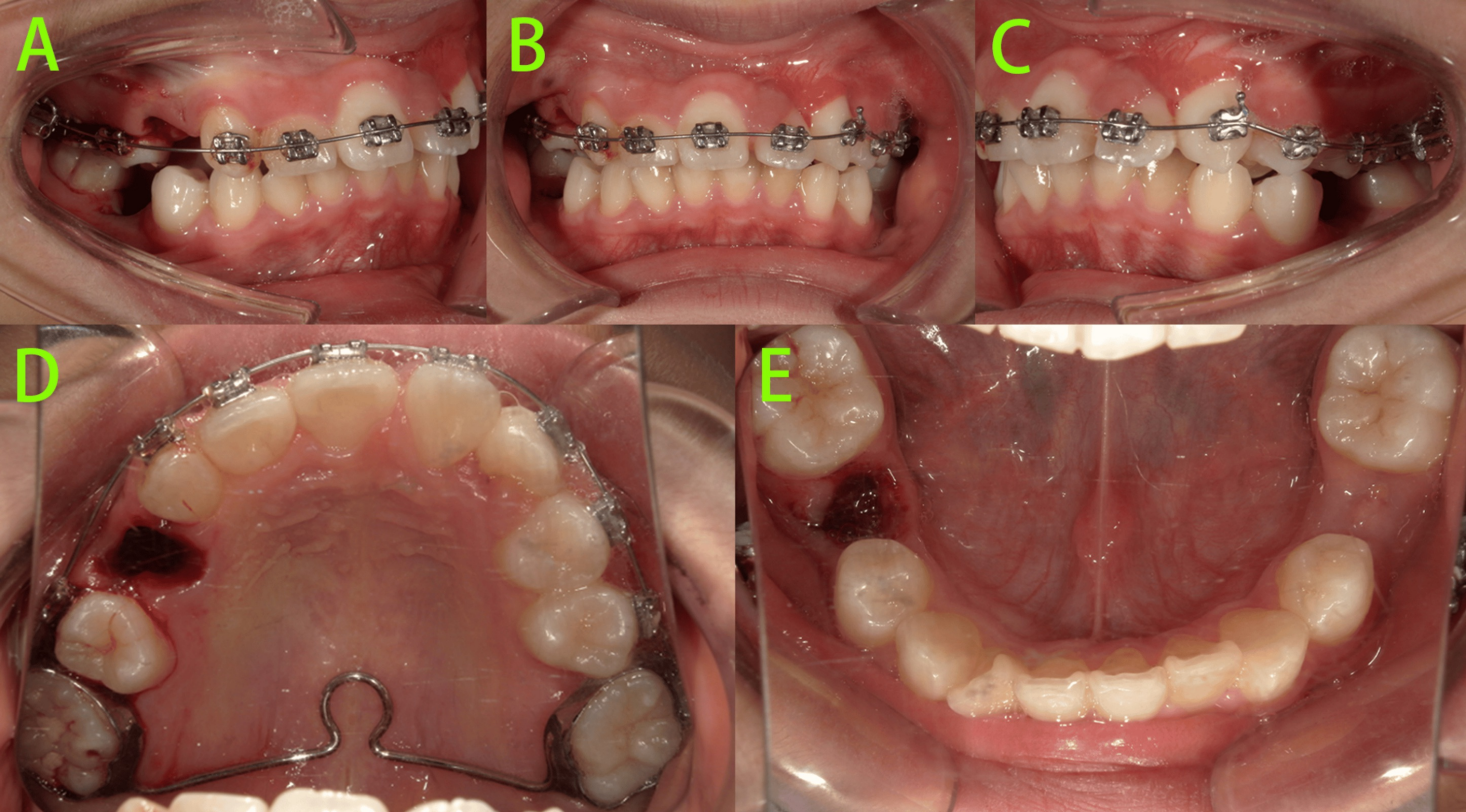
Intraoral photos at 14 months of treatment A-E: Tooth 22 had been successfully mesialized into position, and tooth 23 was nearly aligned.

At 14 months of treatment (Figure 10), tooth 22 had been successfully mesialized into position, and tooth 23 was nearly aligned. Extractions of teeth 14, 35, and 45 were being performed in stages, in preparation for initiating comprehensive fixed orthodontic treatment.

**Figure 11:**
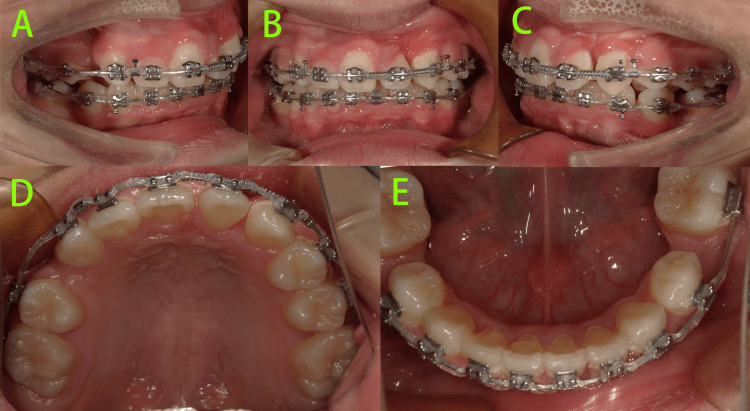
Intraoral photos at 20 months of treatment A-E: Mesiodistal space was being preserved distal to tooth 22 for future composite resin restoration, while awaiting eruption of the second molars, and significant gingival hyperplasia was observed on the mesiolabial aspect of tooth 22.

At 20 months of treatment (Figure 11), the maxillary and mandibular arches had been aligned. Leveling of the curve of Spee was continuing, and the remaining extraction spaces were being closed. Mesiodistal space was being preserved distal to tooth 22 for future composite resin restoration, while awaiting eruption of the second molars. However, significant gingival hyperplasia was observed on the mesiolabial aspect of tooth 22, which was attributed to inadequate oral hygiene and gingival compression during the mesialization of tooth 22.

**Figure 12:**
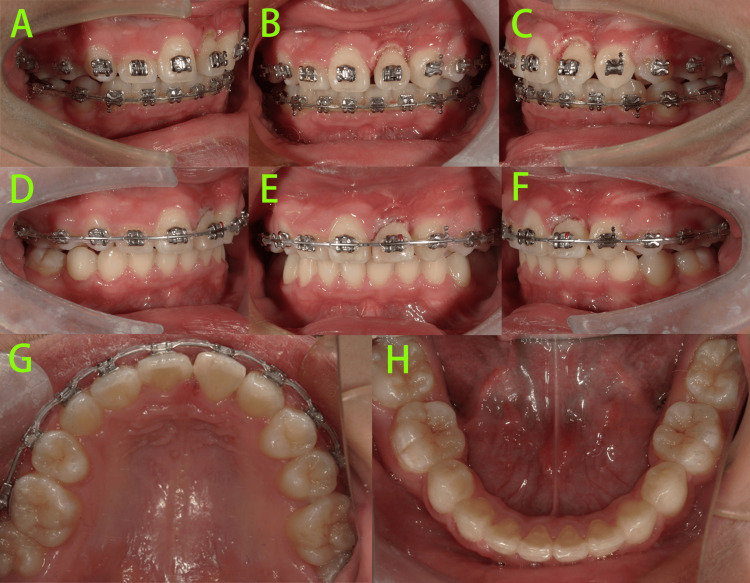
Intraoral photos at 34 months of treatment A-C: Gingivectomy of tooth 22 was performed using electrocautery. D-H: Composite resin restoration of tooth 22 was carried out in the Department of Endodontics to reshape it into the form of a central incisor.

At 34 months of treatment (Figure 12), the maxillary and mandibular arches, including the second molars, had fully erupted and were aligned. The extraction spaces were closed, with bilateral posterior teeth exhibiting a Class I relationship and normal anterior overbite and overjet. First, we perform periodontal scaling. Subsequently, a gingivectomy of tooth 22 was performed using electrocautery. Following this, composite resin restoration of tooth 22 was carried out in the Department of Endodontics to reshape it into the form of a central incisor. After the restoration, a small residual mesiodistal space remained adjacent to tooth 22. The mandibular fixed appliance was then removed, and a mandibular vacuum-formed retainer was delivered, while maxillary space closure was continued to address the remaining gap.

**Figure 13:**
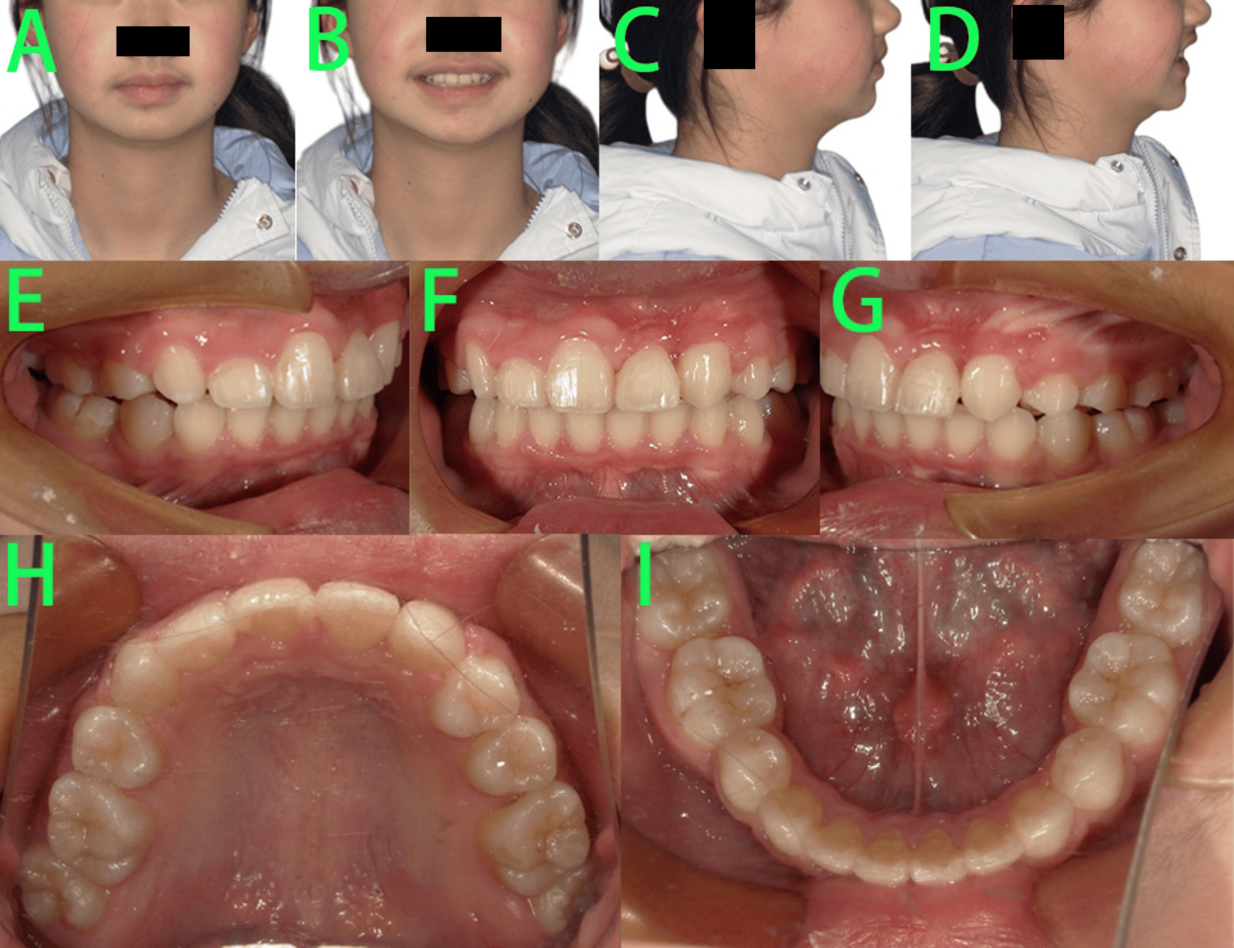
Post-treatment photos A-D: Facial photos revealed the patient's facial profile showed significant improvement in convexity compared to pretreatment, although mandibular retrusion persisted, with a normal lip-to-tooth relationship. E-I: Intraoral photos revealed that the maxillary and mandibular arches were well-aligned with no spaces, tooth 22 had been restored to the morphology of a central incisor.

At 36 months of treatment (Figure 13), comprehensive orthodontic treatment was completed. The patient's facial profile showed significant improvement in convexity compared to pretreatment, although mandibular retrusion persisted, with a normal lip-to-tooth relationship. Intraorally, the maxillary and mandibular arches were well-aligned with no spaces. Tooth 22 had been restored to the morphology of a central incisor. Bilateral molars exhibited a Class I relationship, and anterior overbite and overjet were normal. Mild deviation of the maxillary and mandibular dental midlines was observed. The patient and her parents were highly satisfied with the treatment outcome and agreed to conclude active treatment, transitioning to the retention phase. All fixed appliances were removed. Subsequently, the patient was instructed to wear a vacuum-formed retainer during the day, a Begg retainer for the maxillary arch at night, and a Hawley retainer for the mandibular arch at night. Regular follow-up visits were scheduled.

**Figure 14:**
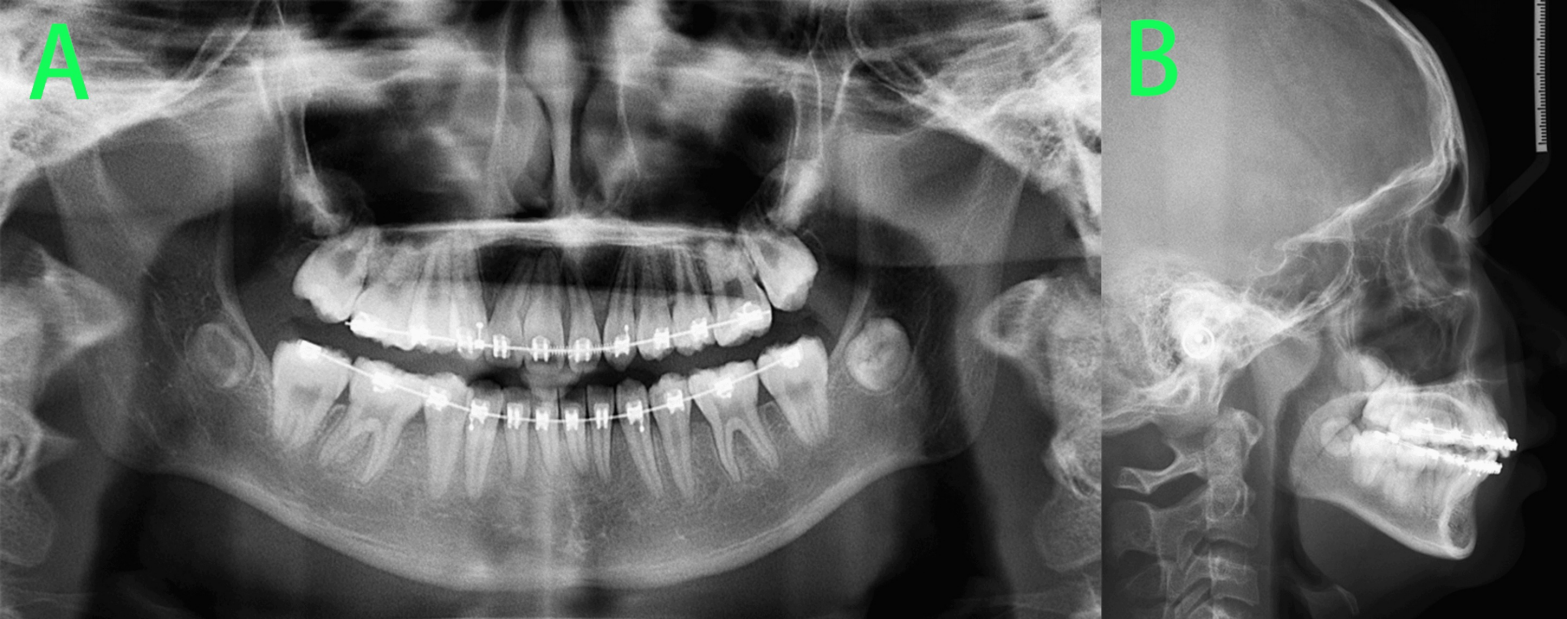
Post-treatment radiographs A: The pre-debonding panoramic radiograph indicated no significant root or alveolar bone resorption in the permanent dentition. B: The lateral cephalometric radiograph revealed an improvement in the skeletal Class II pattern compared to pretreatment, although mandibular retrusion persisted, the maxillary anterior teeth were significantly retracted.

The pre-debonding panoramic radiograph (Figure 14A) indicated no significant root or alveolar bone resorption in the permanent dentition. The lateral cephalometric radiograph (Figure 14B) and cephalometric analysis (Table 2) revealed an improvement in the skeletal Class II pattern compared to pretreatment, although mandibular retrusion persisted. The maxillary anterior teeth were significantly retracted, and no adverse clockwise rotation of the mandible was observed.

**Table 2:** Post-treatment cephalometric measurements

Cephalometric parameters	Pre-treatment	Post-treatment	Reference value
SNA(°)	81.7	81.4	83.0±4.0
SNB(°)	73.7	75.2	80.0±4.0
ANB(°)	8.0	6.2	3.0±2.0
Wits(mm)	6.6	3.9	0.0±2.0
S-Go/N-Me(%)	62.1	62.6	64.0±2.0
FMA(°)	29.2	28.6	26.0±4.0
U1-SN(°)	118.9	96.0	106.0±6.0
IMPA(°)	91.9	101.0	97.0±6.0

**Figure 15:**
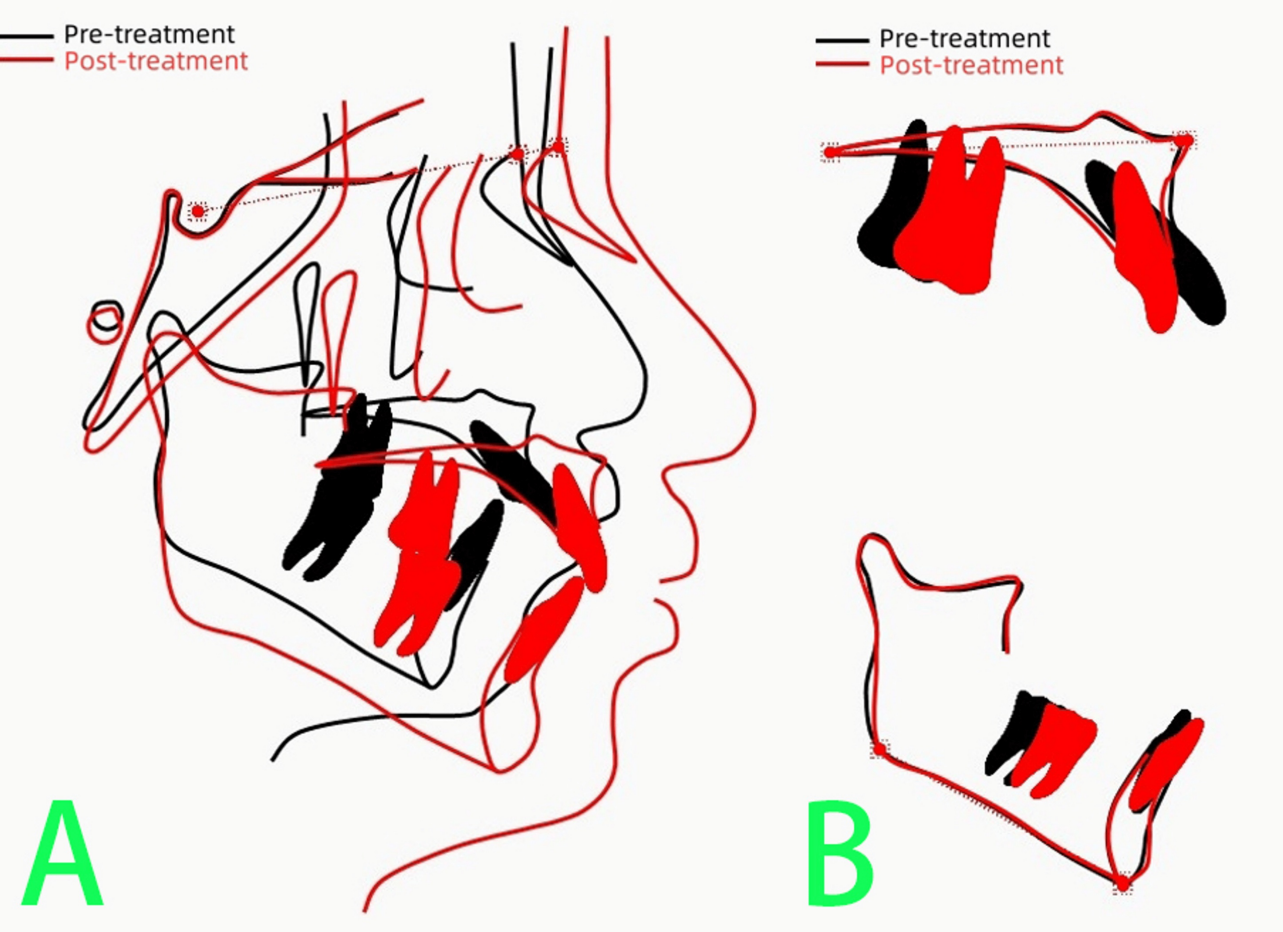
The pre-treatment (black line) and post-treatment (red line) cephalometric superimposition A: Cephalometric superimposition showed continued mandibular growth. B: Cephalometric superimposition showed significant retrusion of the maxillary incisors and mild labial tipping of the mandibular incisors. Image credits: Created by the author Xu S using UCeph software (version 3.1, UCeph Software, Chengdu, China).

The pre-treatment and post-treatment cephalometric superimposition (Figure 15) showed continued mandibular growth, significant retrusion of the maxillary incisors, and mild labial tipping of the mandibular incisors as a compensatory mechanism.

## Discussion

From the perspective of anatomical structures during growth and development, the eruption path of the maxillary canine is more tortuous compared to that of other teeth [18]. For instance, the bony crypt of the permanent maxillary canine is situated at the lateral edge of the piriform aperture, anterior to the maxillary sinus, and below the orbital floor. The position of the tooth germ is located posterior to the root of the deciduous canine. This anatomical location is relatively high. During the entire eruption process, any interference can lead to the ectopic or impaction of the canine. The etiology of impacted maxillary canines mainly includes systemic factors and local factors [19]. Systemic factors may be related to genetic factors; impacted canines often coexist with other hereditary dental abnormalities, such as anomalies in tooth number and morphology, suggesting that these conditions may share a common genetic basis. Systemic factors also include certain systemic diseases such as rickets, ectodermal dysplasia syndrome, cleidocranial dysplasia syndrome, and Down syndrome, etc. Local factors include developmental abnormalities such as an abnormal position of the canine tooth germ, delayed or arrested development of the tooth germ itself, as well as insufficient eruption space, or various mechanical blocking factors, including odontomas and supernumerary teeth. Since the maxillary canine erupts along the root of the lateral incisor after contacting its distal surface, the absence or morphological abnormality of the maxillary lateral incisor may also lead to the ectopic or impaction of the maxillary canine [20]. It is generally believed that insufficient eruption space is an important cause of labial impaction of the maxillary canine [21]. In this case, the panoramic radiograph of the six-year-old patient showed an abnormal direction of the permanent tooth germ of the maxillary canine, along with significant dental crowding, both of which were important contributing factors to the eventual impaction of the canine.

When the canine is severely displaced or its eruption pathway is obstructed, preventing spontaneous eruption, it can be diagnosed as an impacted canine. The general principle is early detection, timely treatment, and efforts to maximize preservation of the affected tooth. Especially for adolescent patients, retaining the canine is of great significance for maintaining the integrity of the dental arch. When assessing the potential prognosis of an impacted canine, for those that may still fail to erupt spontaneously after guidance, timely measures such as space expansion and surgical exposure with orthodontic traction should be taken to avoid complications such as root resorption of adjacent teeth caused by continued displacement of the canine [22,23]. In this case, the patient showed a tendency for canine impaction as early as age six years, but timely treatment was not provided. Five years later, this ultimately resulted in impaction of the canine, accompanied by root resorption of the central incisor. This case once again underscored the importance of early intervention in the management of ectopic eruption and impaction of canines. If the tendency for canine impaction had been identified at age six years, and proactive measures such as the extraction of the deciduous canine in conjunction with space expansion had been taken to guide its eruption, followed by early surgical exposure and orthodontic traction if necessary later, the issue of root resorption of the adjacent tooth might have been avoided.

Currently, imaging examination is the primary method for early prediction and screening of impacted maxillary canines, including panoramic radiographs and CBCT. Panoramic radiography is convenient to perform and involves relatively low radiation exposure, making it the most commonly used method for screening impacted maxillary canines [24]. By observing and measuring the panoramic radiograph, a preliminary assessment can be made regarding the presence of ectopic eruption or impaction of the canine. If further examination is required, CBCT can be used for a more detailed assessment of the impaction position, adjacent relationships, and other factors [25]. In panoramic radiographs, the mesial angulation of the maxillary canine and the degree of overlap between the maxillary canine and the lateral incisor are important predictive indicators for maxillary canine impaction [18,26]. At the same time, special attention should be paid to observing the position and direction of the canine tooth germ, as well as the presence of various pathological factors such as supernumerary teeth, odontomas, and jaw cysts.

The persistence of the root resorption process depends on the activation of osteoclasts by external stimuli. According to the different stimulating factors, root resorption can be classified into: pulp infection-related root resorption, periodontal infection-related root resorption, orthodontic pressure-related root resorption, impacted tooth or tumor pressure-related root resorption, and ankylotic root resorption [27]. External root resorption of adjacent teeth caused by an impacted tooth is often non-infectious in nature, with clinical symptoms typically being unapparent. It is frequently discovered incidentally during imaging examinations. Alternatively, it may be detected only when external resorption progresses, involves the dental pulp, and leads to secondary pulpal infection, at which point patients present with symptoms such as pain or tooth mobility [28]. Currently, the main treatment modalities for this condition include observation, root canal treatment, or extraction [29]. Clinically, the approach usually needs to be determined based on the specific situation. In this case, the root resorption on the labial aspect of the central incisor had extended nearly to the cervical region, rendering the tooth hopeless. Additionally, as the patient presented with skeletal Class II malocclusion and had an indication for extraction camouflage treatment, the central incisor exhibiting root resorption was extracted.

In this case, because the central incisor was extracted, the lateral incisor ultimately replaced the central incisor, and the canine replaced the lateral incisor. Canines play an important role in canine-protected occlusion. Therefore, in this case, careful evaluation was required to determine whether to retain the canine in its normal position and subsequently restore the missing tooth 21 or to move the canine mesially to replace the lateral incisor. The choice between these two treatment options remains somewhat controversial at present [30]. But in general, both options are acceptable for most patients [31]. Undoubtedly, canine substitution involves compromises in functional occlusion, esthetics, and periodontal health [32]. Both canine guidance and group-function guidance occlusions are considered normal; meanwhile, studies have shown that the majority of the population exhibits a group function occlusal scheme [33]. However, one previous study has shown that canine substitution or prosthetic restoration with the canine retained does not differ significantly in terms of the prevalence of occlusion morphology disorders or temporomandibular dysfunction (TMD) [34]. There are differences between the maxillary canines and the lateral incisors in terms of size, shape, shade, etc. It is difficult to achieve acceptable aesthetic results with the canine substitution approach [35]. But on the other hand, anterior implant restoration also presents issues such as vestibular gingival retraction, darkening of the overlying labial gingiva, infraocclusion of the implant, and bone loss around the implants; for adolescent patients, prolonged temporary restoration is also required [30]. Therefore, when selecting the treatment plan for this case, thorough communication was conducted with the patient's parents, and ultimately, the compromised solution of canine substitution was chosen.

Orthodontic treatment for skeletal Class II malocclusion often includes approaches such as functional appliance therapy, extraction, or maxillary molar distalization for camouflage treatment, and combined orthodontic-orthognathic surgical treatment. For adolescents in their peak period of growth and development, functional appliance therapy is a recognized effective treatment method that fully utilizes the mandible's growth potential and stimulates its growth [36]. In this case, the patient manifested as skeletal Class II, mandibular retrognathia, with a normodivergent and average growth pattern. Additionally, being in the pre-peak period of pubertal growth, this made it the optimal indication for functional appliance therapy. However, considering the patient's significant dental crowding and the severe root resorption of the maxillary central incisor, which made it impossible to retain, an extraction camouflage treatment plan was ultimately adopted. Although this approach might not achieve greater mandibular growth or improvement in soft tissue profile, it could avoid the need for future prosthetic restoration and might be more conducive to the patient's long-term oral health.

Although this case ultimately achieved a relatively satisfactory orthodontic camouflage treatment outcome, some limitations remain. Firstly, due to the lateral incisor substituting for the central incisor, there was an inconsistency in the gingival height of the central incisor region. In the later stage, this gingival aesthetic issue could potentially be improved through crown lengthening surgery [37]. Additionally, although the patient's soft tissue profile convexity improved significantly in the end, the mandibular retrognathia remained quite pronounced. It could be considered to combine fixed orthodontic treatment with a fixed mandibular advancement functional appliance, which might have achieved more mandibular growth modification [38]. The torque of the anterior teeth and root parallelism indeed could not be finely adjusted to an optimal state, and the midline was not completely aligned due to asymmetric extractions. Finally, the long-term stability of the canine substituting for the lateral incisor and the premolar substituting for the canine in this case required further follow-up.

## Conclusions

Impacted maxillary canines require timely diagnosis and intervention. Otherwise, impacted canines can lead to complications such as root resorption of adjacent teeth. Although the impacted canine in this case was detected early, timely treatment was not provided, ultimately leading to the complication of root resorption of the central incisor. This once again highlights the importance of timely intervention for impacted canines. When an impacted canine causes severe root resorption of an incisor, whether to extract the incisor with root resorption needs to be carefully evaluated. When the incisor root resorption is too severe to retain the tooth, orthodontic treatment with canine substitution is also an acceptable, compromised treatment option.
